# Urothelium with barrier function differentiated from human urine-derived stem cells for potential use in urinary tract reconstruction

**DOI:** 10.1186/s13287-018-1035-6

**Published:** 2018-11-08

**Authors:** Qian Wan, Geng Xiong, Guihua Liu, Thomas D. Shupe, Guanghui Wei, Deying Zhang, Dan Liang, Xiongbing Lu, Anthony Atala, Yuanyuan Zhang

**Affiliations:** 1 Wake Forest Institute for Regenerative Medicine, Wake Forest School of Medicine, Winston-Salem, NC USA; 2 State Key Laboratory of Ophthalmology, Zhongshan Ophthalmic Center, Sun Yat-Sen University, Guangzhou, China; 3 Ministry of Education Key Laboratory of Child Development and Disorders, Chongqing Key Laboratory of Child Urogenital Development and Tissue Engineering, Department of Urology, Children’s Hospital of Chongqing Medical University, Chongqing, China; 4 Reproductive Medicine Research Center, The Sixth Affiliated Hospital of Sun Yat-Sen University, Guangzhou, China; 5 Department of Urology, The Second Affiliated Hospital at Nanchang University, Nanchang, China

**Keywords:** Urothelium, Urine-derived stem cells, Barrier function, Tight junctions, Bladder diseases, Tissue engineering

## Abstract

**Background:**

Autologous urothelial cells are often obtained via bladder biopsy to generate the bio-engineered urethra or bladder, while urine-derived stem cells (USC) can be obtained by a non-invasive approach. The objective of this study is to develop an optimal strategy for urothelium with permeability barrier properties using human USC which could be used for tissue repair in the urinary tract system.

**Methods:**

USC were harvested from six healthy adult individuals. To optimize urothelial differentiation, five different differentiation methods were studied. The induced cells were assessed for gene and protein expression markers of urothelial cells via RT-PCR, Western blotting, and immunofluorescent staining. Barrier function and ultrastructure of the tight junction were assessed with permeability assays and transmission electron microscopy (TEM). Induced cells were both cultured on trans-well membranes and small intestinal submucosa, then investigated under histology analysis.

**Results:**

Differentiated USC expressed significantly higher levels of urothelial-specific transcripts and proteins (Uroplakin III and Ia), epithelial cell markers (CK20 and AE1/AE3), and tight junction markers (ZO-1, ZO-2, E-cadherin, and Cingulin) in a time-dependent manner, compared to non-induced USC. In vitro assays using fluorescent dye demonstrated a significant reduction in permeability of differentiated USC. In addition, transmission electron microscopy confirmed appropriate ultrastructure of urothelium differentiated from USC, including tight junction formation between neighboring cells, which was similar to positive controls. Furthermore, multilayered urothelial tissues formed 2 weeks after USC were differentiated on intestine submucosal matrix.

**Conclusion:**

The present study illustrates an optimal strategy for the generation of differentiated urothelium from stem cells isolated from the urine. The induced urothelium is phenotypically and functionally like native urothelium and has proposed uses in in vivo urological tissue repair or in vitro urethra or bladder modeling.

**Electronic supplementary material:**

The online version of this article (10.1186/s13287-018-1035-6) contains supplementary material, which is available to authorized users.

## Background

Urothelial cells (UC) are classified as transitional epithelium, and they cover almost the entire luminal surface of the urinary tract. This includes the renal pelvis, ureters, bladder, and the proximal segment of the urethra. Urothelium provides a robust permeability barrier across the urinary tract. This barrier function is dependent on tight-junction complexes that limit the transfer of ions and solutes across the urothelium. In addition, surface glycans, specialized lipid molecules, and uroplakins at the apical surface further reduce the permeability of the urothelium [1]. The barrier function of the urothelium protects underlying tissue from toxic components of urine. Formation and degeneration of the urothelium are critical to inhibit urethral stricture development. In addition, an intact urothelium prevents bladder detrusor muscle over-activity [2], inflammation, and fibrosis. Compromised urothelium leads to several common urologic diseases such as recurrent urinary tract infection, urethral injuries or stricture, interstitial cystitis, overactive bladder, and bladder cancer.

Bioengineered urothelium would provide a valuable tool for both the development of engineered urothelium for urological reconstruction and the study of urothelial dysfunction. Currently, few in vitro urothelial models exist for the development of drugs intended to treat various urologic disorders in the low urinary tract. Thus, there is an urgent need for the generation of engineered urothelium for urinary tract tissue repair. Our group has developed technologies for the generation of engineered multilayer urothelial sheets with barrier function [3–8]. These constructs are currently generated using differentiated cells from healthy urothelial tissue obtained from different species, including rat, pig, and human [3–8]. However, when healthy urothelial tissue is not available, stem cells may be used as an alternative cell source. Mesenchymal stem cells (MSC) derived from bone marrow [9, 10] or adipose tissue [11] maintain some capacity for urothelial cell differentiation. In addition, embryonic stem cells (ESC) [12] and induced pluripotent stem cells (iPSC) [13] have successfully been differentiated into urothelial cells. Despite advances in the controlled differentiation of these progenitor cells, each type has drawbacks and limitations. MSC have limited urothelial differentiation potential, harvesting cells results in donor site morbidity, and there remain concerns regarding the oncogenic potential of iPSC-derived cells as well as persistent ethical issues regarding the use of ESC. An alternative, autologous stem cell source for the generation of UC would benefit the fields of drug development, personalized medicine, and regenerative medicine.

Our previous studies have demonstrated that human urine-derived stem cells (USC) shed from the urinary tract possess beneficial regenerative properties, including robust proliferative potential and multi-potent differentiation potential [14–16]. There are several advantages of human USC over other stem cells. USC can be easily obtained from healthy individuals or patients by non-invasive and low-cost procedures that generate high-quality cells that can be expanded extensively [14, 17–19]. Up to 140 USC clones per 24-h urine collection were consistently obtained from a single healthy individual [20]. Thus, a 24-h urine sample can provide > 1 × 10^8^ cells over three passages, a number sufficient for a majority of the intended applications. In addition, cell viability is preserved during isolation [20, 21] as the method used does not require enzymes (such as collagenase) for tissue dissociation. Furthermore, no evidence for oncogenic potential in human USC has been identified over several in vivo studies [14, 17, 22].

Our previous studies have revealed that USC can efficiently give rise to cells expressing urothelial cell markers; however, the barrier function and cellular structure of urothelial cells cultures generated from USC have not yet been investigated [13–16]. The purpose of this study was to optimize a strategy to induce human USC differentiation into functional urothelial cells with barrier function and appropriate cellular 3D architecture. These cells would represent a powerful tool for urological tissue research and urological regenerative medicine.

## Methods

### Cell isolation, culture and differentiation

Collection of human urine and bladder tissues in this study was approved by the Wake Forest University Health Sciences Institutional Review Board. In total, 28 urine samples were freshly collected from 6 healthy male individuals (28–55 years old). USC were isolated and cultured as previously described [19]. Briefly, urine was centrifuged at 500 g for 5 min and cell pellets were suspended in a mixed medium composed of embryo fibroblast medium (EFM) and keratinocyte serum-free medium (KSFM) (EFM-KSFM, 1:1 ratio) with 10% fetal bovine serum (FBS). The cells were cultured in 24-well plates for 3–5 days, at which point USC clones appeared. When reaching 70–80% confluence (*p0*), USC were passaged to six-well plates (*p1*). USC at *p2–5* were used for all experiments as described below. Human smooth muscle cells (SMC) and human UC were used to provide conditioned medium, and normal UC were used as a positive control. Both cell types were isolated from human bladder biopsies or ureteral tissue from donated kidneys [7]. SMC were cultured in Dulbecco’s modified Eagle’s medium (DMEM) with 10% FBS and UC were cultured in KSFM with supplements. For all experiments, UC and SMC were used before *p3*.

### Flow cytometry

To evaluate stem cell surface markers, cultured USC at *p2* were stained with specific anti-human antibodies: CD45-FITC, CD31-FITC, CD73-PE, CD90-FITC, CD105-PerCP-Cy™5.5, CD34-FITC, CD44- FITC and CD146-PE. Briefly, following trypsinization, cells (5 × 10^5^) were re-suspended in ice-cold phosphate buffered saline (PBS) containing 1% bovine serum albumin (BSA). Fluorochrome-conjugated antibodies were added to cells in 50 ml PBS containing 3% BSA and incubated on ice for 30 min in the dark. IgG1-PE, IgG1-FITC, IgG2b-FITC, and IgG1-PerCP-Cy™5.5 conjugated isotype control antibodies (BD Pharmingen™, Sparks, MD) were used to determine background fluorescence. Cells were then washed twice in wash buffer, passed through a 70-μm filter, and analyzed by flow cytometry (FACSCalibur BD Biosciences, Franklin Lakes, NJ).

### Optimization of urothelial differentiation methods

To efficiently induce USC differentiation into urothelial cells, differentiation methods were optimized under several induction conditions (Table 1), in both dynamic and static cultures for different culture periods (1, 2, or 3 weeks). Assessment of barrier function was accomplished by evaluation of tight junction formation (Western blotting, real-time PCR, immunofluorescence), transmission electron microscopy, and fluorescent dye exclusion.

**Table 1:** Research design for optimization of urothelially differentiated human USC

Group (G)	Dynamic culture		Static culture	
	Single seeding	Triple seeding	Single seeding	Triple seeding
G1 USC	USC (5 × 10^5^ cells/well) as negative control			
G2 UC	UC (5 × 10^5^ cells/well) cultured in KSFM as positive control			
G3 USC + UC/CM	USC (5 × 10^5^ cells/well) cultured with conditioned Media from UC culture			
G4 USC + EGF	USC (5 × 10^5^ cells/well) cultured in induce Media with EGF (30 ng/ml)			
G5 USC + SMC/CM	USC (5 × 10^5^ cells/well) cultured with EGF (30 ng/ml) and conditioned medium from SMC culture (1:1)			

Abbreviations: *USC* urine-derived stem cells, *UC* urothelial cells, *SMC* smooth muscle cells, *CM* conditioned medium, *UC/CM* urothelium-conditioned medium, *SMC/CM* Smooth muscle cell-conditioned medium, *EGF* epidermal growth factor

Conditioned medium was collected 8–12 h after cultured UC or SMC (at p3), respectively. Centrifuged at 1500 RPM for 5 min, the supernatant was filtered with a microfilter (pore size of 0.22 μm, Corning, Tewksbury, MA) to void cell contamination. For urothelial induction, USC were firstly seeded in six-well plates at 5 × 10^4^ cells /cm^2^ under ordinary stem cell media [14]. To evaluate urothelial induction conditions, USC were treated with three different types of differentiation media, compared to positive (UC) and negative (non-induced USC) controls, see Table 1. To determine the effect of secretomes of urothelial cell culture on differentiation of USC, conditioned medium from UC culture mixed with EFM-KSFM (1:1), compared to a standard induction method [14, 21], i.e., KSFM containing epidermal growth factor (EGF) at 30 ng/ml. In addition, to evaluate the effect of epithelial-stromal interaction, conditioned medium from SMC culture on urothelial induction of USC will be tested when mixed with KSFM (1:1).

To evaluate the impact of 3D dynamic culture on cellular growth and differentiation, cells of each group were seeded on the culture plates for 6 h and then loaded onto an orbital shaker (Belly Dancer, Stovall, Greensboro, NC) at 40 revolutions per minute (rpm), compared to static culture at different time points (1, 2, or 3 weeks, see below), as described previously [22]. The medium was replaced every 3 days. USC, UC, and SMC controls were cultured under the same conditions described above.

To determine the temporal kinetics of urothelial differentiation, USC and the cells at control groups were cultured in each condition for 1, 2, and 3 weeks.

### Permeability determinations

Induced USC were cultured on Falcon® 23.1 mm Permeable Support with 0.4 μm Translucent High-Density PET Membrane (Corning, New York, USA), as previously reported with minor modifications [23]. Briefly, the upper inserts were coated with collagen-IV (3 μg/cm^2^), air dried in a laminar hood, and sterilized by a 70% ethanol rinse. The ethanol was allowed to evaporate completely before the inserts were used. To assess the barrier function of UC-induced USC, cells (1 × 10^5^/cm^2^) were plated in 1.5 ml of 1 mg/ml tracer-containing medium (FITC-dextran, 4 kDa, Sigma, FD4) in the insert (top chamber) and 3 ml tracer-free medium in the bottom well. Phenol-free medium was used to avoid interference of the indicator in the assay. The media were supplemented with 2 mM CaCl_2_ solution 24 h before the tracer was added. Three hours after the tracer was added, 100 μl media aliquots from the bottom wells were collected for fluorescence measurements (excitation at 490 nm and emission at 520 nm). Tracer diffusion across the cell layers was calculated by measuring the fluorescent intensity of FITC-dextran in the lower chamber.

### Real-time PCR

Total mRNA was extracted from cell pellets based on culturing and grouping above using the RNA isolation kit (5Prime, Gaithersburg, MD). According to the manufacturer’s instructions, 5 μg of RNA was converted to cDNA in a reaction containing primers, nucleotides, and reverse transcriptase enzyme using a high-capacity cDNA reverse transcription kit (Applied Biosystems, Foster City, CA, USA). The cDNA was used for real-time analysis along with Taqman Universal PCR master mix and gene expression probes. The assay was performed using a 7300 Real-time PCR system (Applied Biosystems, Foster City, CA, USA). The primer sequences used in this study are listed in Additional file 1: Table S1.

### Western blotting

Cells were harvested from six-well plates and lysed for Western blotting analysis as previously described [14]. Proteins were separated by 6–10% sodium dodecyl sulfate-polyacrylamide gel (10–50 μg/lane) and then transferred to nitrocellulose membrane. The membrane was probed with primary antibodies at 4 °C overnight and then incubated with secondary antibodies at room temperature for 1 h. Protein hybridization was detected by using the enhanced chemiluminescent assay. Images were captured by a Fujifilm imaging system (LAS 3000). For quantification of signal, the Multi Gauge V3.0 software from Fujifilm was used and the value was presented as relative density to β-actin. The primary antibodies used in this study are listed in Additional file 2: Table S2.

### Immunofluorescence

USC were induced following 14 days in dynamic culture conditions, sub-cultured, and then seeded onto chamber slides (Thermo Fisher Scientific Inc., Waltham, MA) and incubated overnight. Cells were fixed with 4% paraformaldehyde, permeabilized with 0.2% Triton for 3 min, and blocked with the blocking buffer (1× PBS/5% normal goat serum/0.3% Triton™ X-100, Cell Signaling Technology, Boston, MA). Urothelial-specific markers (i.e., Uroplakin Ia, Uroplakin III, and CK20, AE1/AE3) and intercellular junction markers (i.e., ZO1, ZO2, E-cadherin, and Cingulin) were used to characterize differentiated USC. An appropriate secondary antibody conjugated to fluorescein isothiocyanate was used. The cells were mounted in a diamidino-2-phenylindole (DAPI, Sigma-Aldrich, St Louis, MO, USA)-containing mount (Vector, Burlingame, CA) for staining nuclei.

All stained sections were evaluated by a single, experienced individual in a double blind manner. All antibodies used for immunofluorescence are listed in Additional file 2: Table S2. To determine the portion of cells expressing positive markers, the number of positive cells were counted and related to the total number of cell nuclei per image. Images were captured from five fields per well at 40× and 200× magnifications with a Zeiss inverted fluorescent microscope (model Axiovert 200 M), filters suitable for DAPI (blue) or fluorescein (green), and a CCD camera from QImaging (model Retiga 2000RV).

### Transmission electron microscopy

The ultrastructure of tight junctions between UC-differentiated USC was examined by transmission electron microscopy (TEM) 14 days after induction. The induced USC seeded onto transwell membranes were fixed and sectioned according to standard procedures. Briefly, the cells were fixed in 2.5% glutaraldehyde, then post-fixed with 1% osmium tetroxide, dehydrated in graded alcohols, embedded in Spurr’s resin (Polysciences, Warrington, PA), and cut into 80-nm sections using a Reichert-jung Ultracut E ultramicrotome. The sections were viewed and captured using a Tecnai Spirit BioTwin transmission electron microscope (FEI, Hillsboro, OR).

### Generation of small intestinal submucosa matrix

A segment of fresh porcine small intestine was obtained from a local slaughter house. Small intestinal submucosa (SIS), a natural collagen-based matrix, was acquired after the mucosa and serosa of intestinal tissue were removed manually and washed in distilled water. For decellularization [3], SIS specimens were immersed in 5% peracetic acid (PAA) for 30 min and then washed in distilled water on a rotary shaker at 200 rpm. SIS material was soaked in 1% Triton X-100 at 4 °C for 2 days and washed in distilled water for 1 day. For disinfection, SIS samples were oxidized in 5% PAA and 20% ethanol for 2 h. Finally, samples were rinsed three times in distilled water and stored in distilled water at 4 °C until needed. SIS scaffolds were firmly secured over a sterile silicone insert for ease of cell seeding.

### Multilayer urothelium formation in vitro

To optimize the structures of multilayer urothelium, three different seeding methods were used for USC to seed onto trans-well membranes: (i) 1 × 10^5^ /cm^2^ plated at one time (single seeding), (ii) a total of 3 × 10^5^ cells/cm^2^ plated in 1 × 10^5^ cells/cm^2^ density over 3 days (triple seeding), (iii) 3 × 10^5^ cells/cm^2^ plated at one time (single seeding). Certain amounts of cells were detached during culture with the last seeding method (i.e., 3 × 10^5^ cells/cm^2^ on single seeding); therefore, the first two seeding methods were used in the rest of study. These cells were induced in urothelial-conditioned medium under dynamic culture conditions for 14 days. Culture plates were placed on a rotator in an incubator at 40 rpm, 24 h after seeding. Cell proliferation of UC-induced USC was measured at 490 nm on days 0, 2, 4, 6, 8, 10, 12, and 14 using an MTT assay (Promega, Madison, WI, USA).

To determine the impact of natural collagen matrix on multilayer urothelium formation by induced USC, cells at were cultured on SIS scaffolds compared to cells on polyester membrane in transwell. Induced USC at 1 × 10^5^/cm^2^ were seeded onto the matrix each day over the first 3 days under static culture and then cultured in dynamic culture for extra 11 days. The cell-seeded SIS and cell transwell membrane constructions were cut into 1-cm^2^ pieces for histological analysis.

### Histology and immunohistochemistry

Cell-seeded matrix samples were analyzed using immunohistochemistry. The samples were fixed in 10% buffered formalin and embedded in paraffin. After deparaffinization and high temperature citrate-based antigen retrieval, the slides were blocked with BLOXALL solution (Vector Laboratory Inc., Burlingame, CA, USA) and 5% goat serum. The slides were then washed in PBS and incubated in anti-cytokeratin antibodies (AE1/AE3, GA053, Dako, CA, USA) and incubated with the secondary antibody, i.e., Biotinylated Goat Anti-Mouse IgG Antibody (Vector Laboratory Inc., Burlingame, CA, USA) diluted at 1:400 for 30 min at room temperature. Finally, after incubation with the ABC reagent, i.e., Vectastain ABC Kit (Vector Laboratory Inc., Burlingame, CA, USA), slides were treated with DAB substrate, i.e., ImmPACT DAB Substrate Kit (Vector Laboratory Inc. Burlingame, CA, USA) and counterstained with hematoxylin.

### Statistical analysis

The values are expressed as means and standard errors of the mean (SEM), with *n* = 3 or 6. All results were reproduced in experiments at least three times. Statistical analyses were performed by using one-way ANOVA. Student-Newman-Keuls post hoc test was used for multiple comparisons. SPSS16.0 software was used for analyses. Differences were considered statistically significant at *p < 0.05*.

## Results

**Fig. 1:**
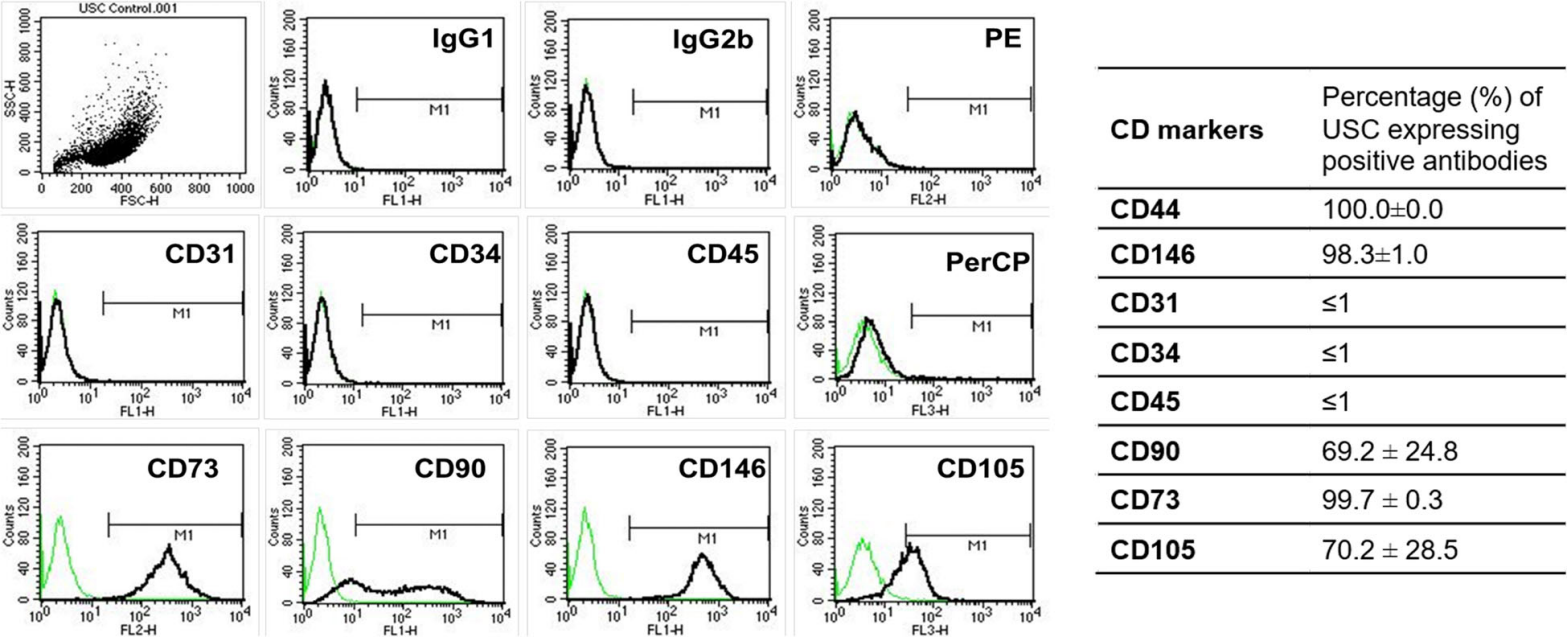
Cell surface markers and morphology of USC and UC-induced USC. Human USC expressed MSC surface marker profiles (CD73, CD90, CD 105, and CD146), but not hemoprotein stem cell markers (CD 31, CD34, and CD45) assessed by flow cytometry

USC adopted a “rice-grain” like appearance 2–3 days after initial seeding. These cells subsequently formed clones over 4–6 days. Fluorescence activated cells sorting (FACS) analysis showed that USC consistently expressed stem cell surface markers (CD73, CD90, CD105) (Fig. 1), but did not express hematopoietic stem cell markers (CD31, CD34, CD45) or endothelial cell markers (CD31) as described previously [13–16]. The induced USC converted to cobblestone morphology, which appeared similar to normal UC. When cultured under conditioned medium, the cells became smaller and more densely packed, as compare to non-induced USC.

**Fig. 2:**
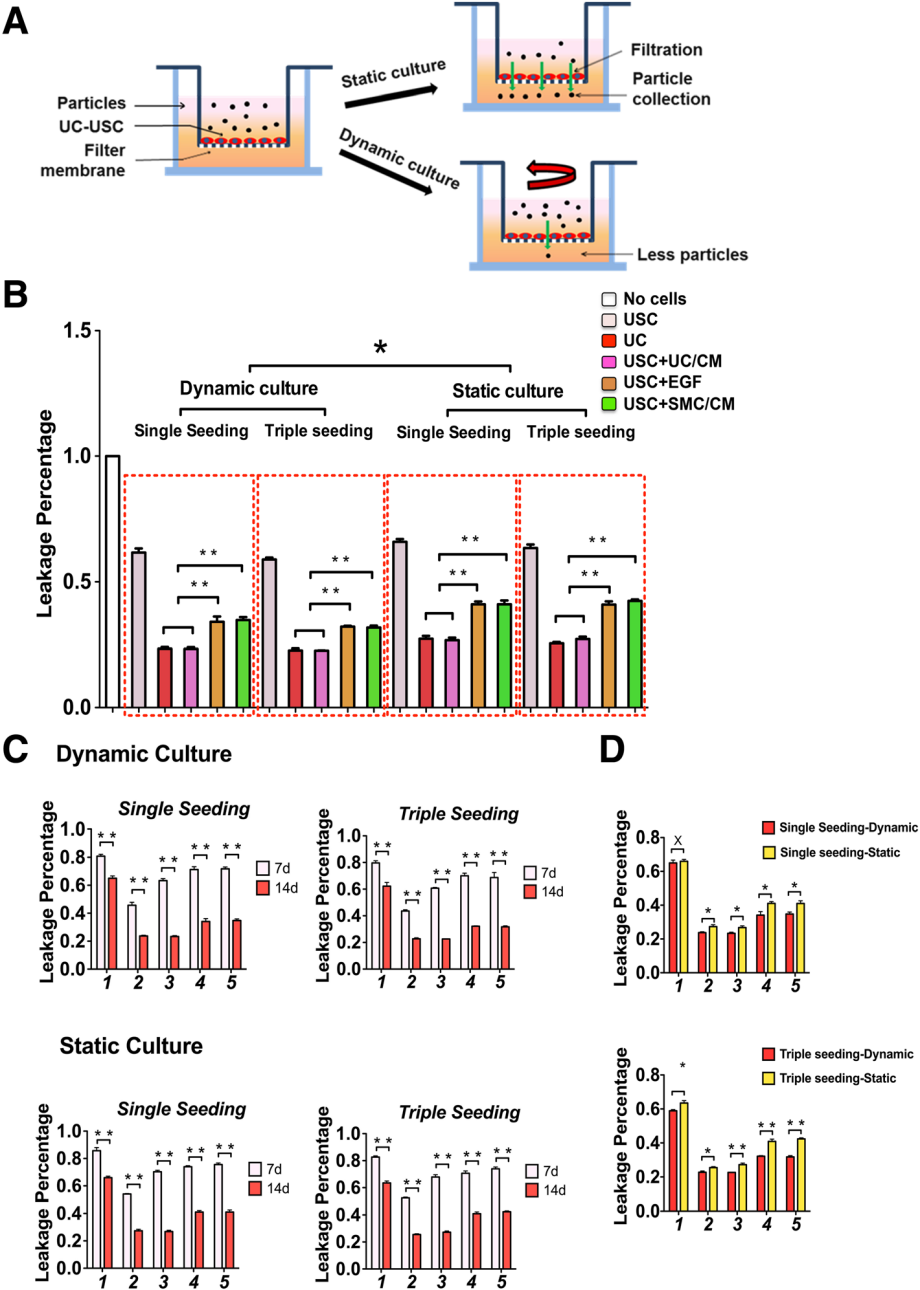
Permeability barrier function test of urothelial differentiation of USC. **a** In vitro permeability test model of UC-induced USC in dynamic culture condition vs. static culture condition. **b** Urothelial induction of USC in different conditions of differentiation culture for 2 weeks. **c** Effect of time-dependent induction on urothelial differentiation of USC on weeks 1 and 2, respectively. **d** Impact of dynamic culture on permeability barrier function of USC 2 weeks after urothelial induction. Notes: Foot numbers in graphs **c** and **d** represent: (1) USC, (2) UC, (3) USC + UC/CM, (4) USC + EGF, (5) USC + SMC/CM. Abbreviations: USC = urine-derived stem cells, UC = urothelial cells, SMC = smooth muscle cells, CM = conditioned medium, UC/CM = urothelium-conditioned medium SMC/CM = smooth muscle cell-conditioned medium EGF = epidermal growth factor. Single seeding = seeding cells only once, Triple Seeding = seeding cells each at first 3 days

In the present study, we compared the addition of urothelial-conditioned medium with or without dynamic flow culture (Fig. 2a), in combination with convention methods (i.e., EGF and static culture) for directing the differentiation of USC to urothelial cells. Barrier function testing is one of most important and effective parameters for the evaluation of urothelial cell differentiation. As such, in vitro assays using a fluorescent tracer on USC cultured on transwell membranes demonstrated a 60% reduction in passage of the tracer across the urothelium over 3 h, as compared to undifferentiated USC. Urothelial cells differentiated in this conditioned medium form continuous cell layers that possess barrier function similar to that of normal urothelium (Fig. 2b). Importantly, the combination of urothelial-conditioned medium and dynamic culture significantly increased urothelial differentiation and barrier function, similar to that of normal urothelium, as compared to other methods, including EGF only or bladder SMC-conditioned medium (Fig. 2b–d, Additional file 3: Table S3, Additional file 4: Table S4) at 2 weeks post-induction (*p* < 0.01).

Temporal analysis of the data indicated that urothelial cell differentiation progresses in a time-dependent manner. Over a 2-week induction period using a combination of conditioned medium and dynamic culture, progressively greater levels of functional proteins and tight junctions formed (Fig. 2c). By comparison, a 1-week induction using EGF produced only modest expression of urothelial cell-specific markers (*p* < 0.01). In addition, dynamic culture alone was sufficient to enhance urothelial transcript and protein marker expression and promoted formation of multilayered urothelial structures (Fig. 2d).

**Fig. 3:**
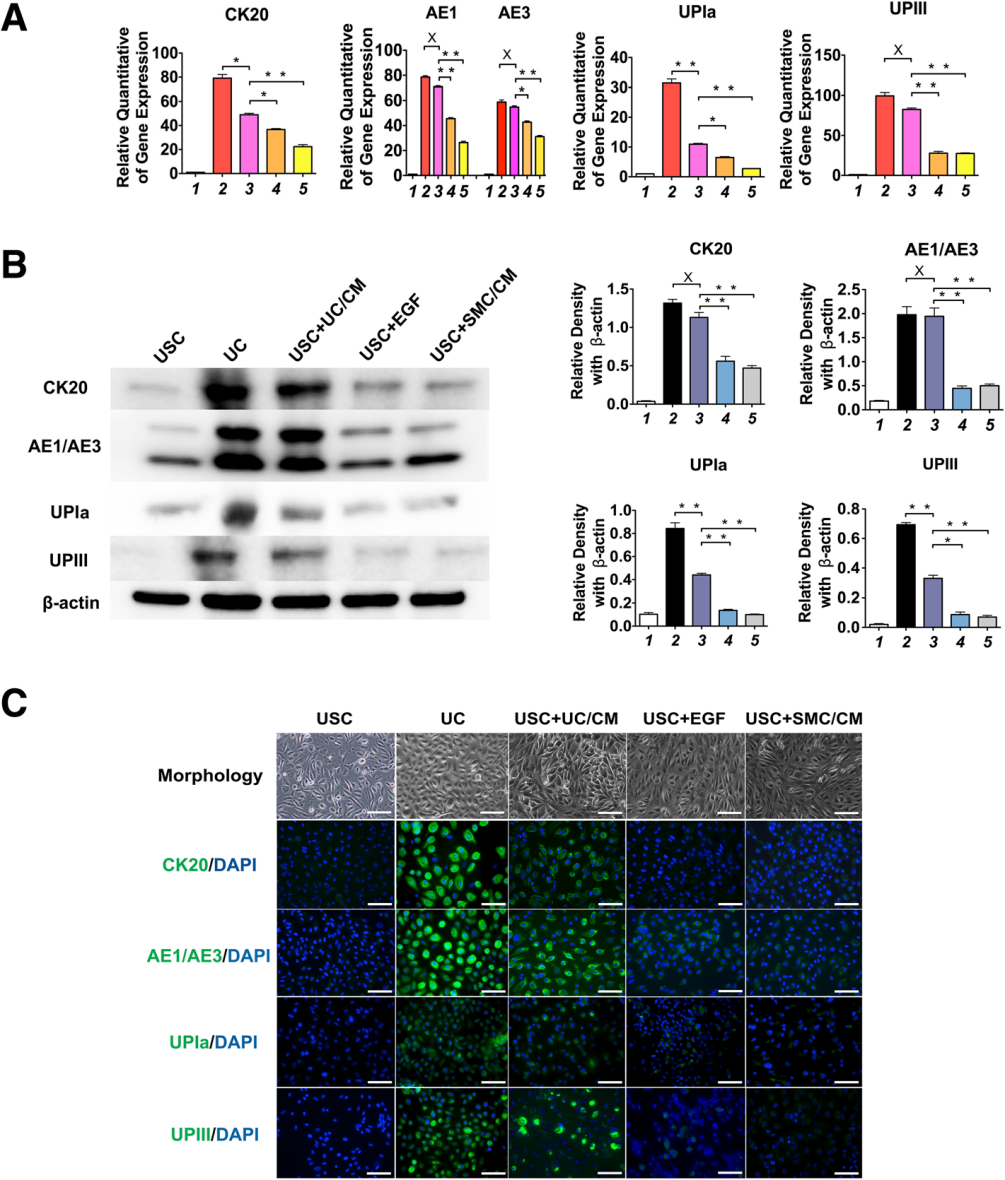
UC-induced USC expressing urothelial cell gene and protein markers. Expression level of urothelial cell transcripts and protein markers (Uroplakin Ia/III, AE1/AE3, and CK20) under different induction culture conditions assessed by: **a** real-time PCR, **b** Western blotting, and **c** immunofluorescence staining 14 days after urothelial differentiation. Cell morphology of USC, UC, and induced USC are also illustrated. Notes: Foot numbers in graphs (**a**) and (**b**) represent: (1) USC, (2) UC, (3) USC + UC/CM, (4) USC + EGF, (5) USC + SMC/CM. Abbreviations: UPIa = Uroplakin Ia, UPIII = Uroplakin III. USC = urine-derived stem cells, UC = urothelial cells, SMC = smooth muscle cells, CM = conditioned medium, UC/CM = urothelium-conditioned medium SMC/CM = Smooth muscle cells conditioned medium EGF = epidermal growth factor. Scale bar = 50 μm

Our current data demonstrate that the optimal material for inducing urothelial cell differentiation from USC is a urothelial-conditioned medium. Immunostaining and Western blotting showed that uroepithelial transcript and protein marker expression by USC for all urothelial cell subtypes (i.e., Uroplakin Ia, Uroplakin III, CK20 for superficial or umbrella cells; AE1/AE3 for all urothelial cells) are significantly increased following combined use of urothelial-conditioned medium and dynamic culture, as compared to other induction conditions (*p* < 0.01, Fig. 3a–c). Cytometer analysis showed that 72.6% of induced USC and 94.5% of UC expressed AE1/AE3, as compared to 15.1% of EGF-induced USC, 19.4% SMC/CM-induced USC, and 18.2% of USC (Additional file 3: Table S3). Dynamic culture conditions significantly improved the barrier function of USC-derived urothelial cells, as evidenced by increased uroplakin expression (Fig. 3a–c). This effect was observed for both native urothelial cells and urothelium differentiated from USC.

**Fig. 4:**
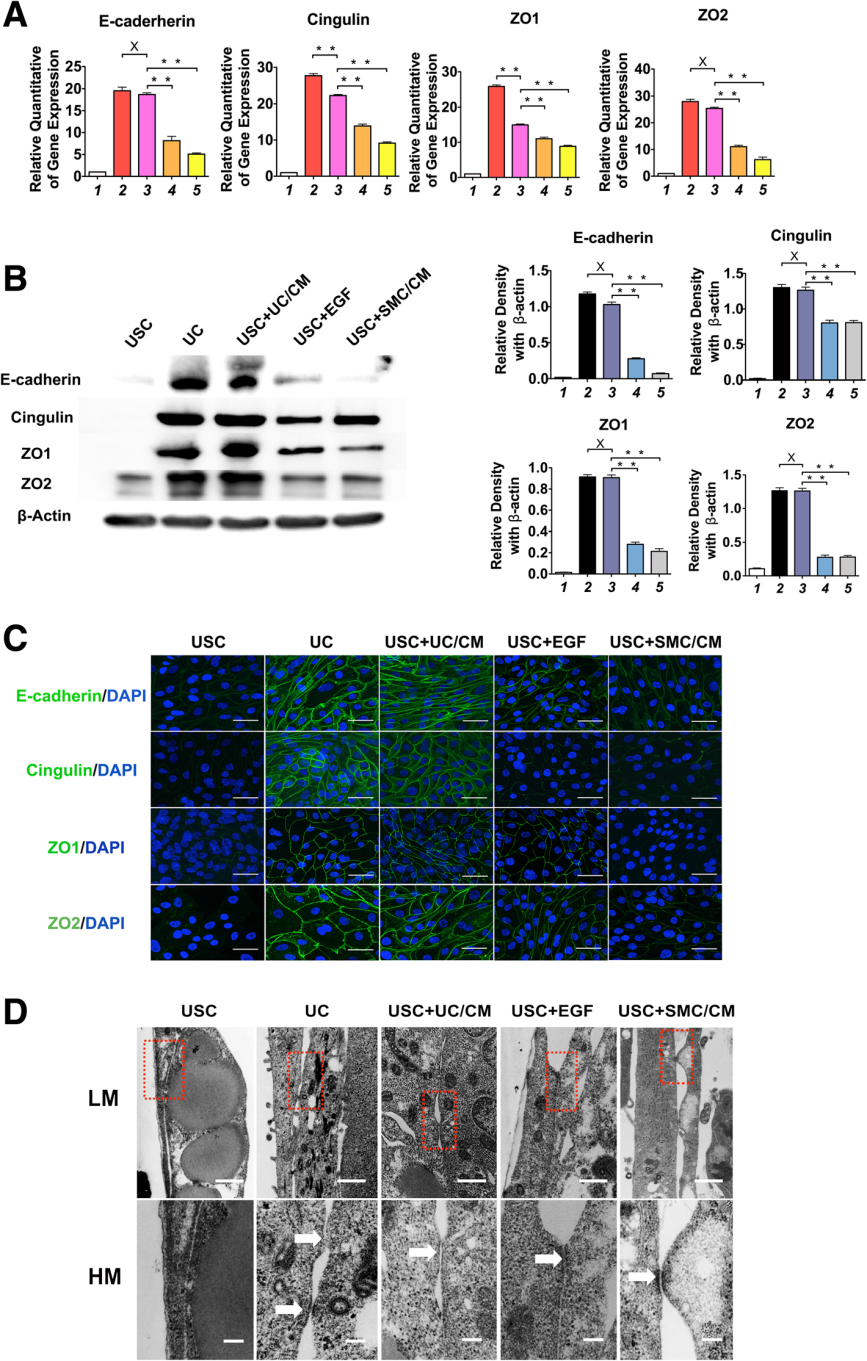
UC-induced USC expressed gene and protein markers of tight junction markers 14 days after differentiation. Tight junction markers (E-cadherin and Cingulin, ZO1, ZO2) displayed on cell membrane boundaries between cells in the urothelially induced USC and UC, but not in USC, assessed by **a** real-time PCR, **b** Western blotting, and **c** immunofluorescent staining assessed under confocal microscope. Scale bar = 40 μM. **d** Tight junctions or desmosomes between neighboring cells in the induced USC and UC groups, but not in non-induced USC, examined by transmission electron microscopy. LM scale bar = 500 nm, HM scale bar = 100 nm. Notes: Foot numbers in graphs **a** and **b** represent (1) USC, (2) UC, (3) USC + UC/CM, (4) USC + EGF, (5) USC + SMC/CM. Abbreviations: USC = urine-derived stem cells, UC = urothelial cells, SMC = smooth muscle cells, CM = conditioned medium, UC/CM = urothelium conditioned medium SMC/CM = Smooth muscle cells conditioned medium EGF = epidermal growth factor. LM = low magnitude, HM = high magnitude

Similarly, several tight junction gene and protein markers (ZO-1, ZO-2, Cingulin, and E-cadherin) are significantly higher with both of UC and USC induced by UC-conditioned medium treatment, as compared to other treatments. This is illustrated by immune-fluorescence staining and Western blotting analysis. Interestingly, high concentrations of EGF (30 ng/ml) in the culture medium also promoted formation of tight junctions, whereas culturing with SMC-CM had little effect (Fig. 4 a–c, Additional file 4: Table S4). Tight junction formation by urothelial cells in vitro requires a confluent cell layer. The structural integrity of tight junctions is one of the most critical components of normal urothelial barrier function. As a barrier, tight junctions functionally block the passing of molecules and ions through the channel between plasma membranes of neighboring cells. By using TEM, the ultrastructure of tight junctions and desmosomes between neighboring cells were identified clearly on both low- and high-definition views of UC and induced USC, including UC-conditioned medium, EGF, and SMC medium treatment; however, they were not found on non-induced USC (Fig. 4d).

**Fig. 5:**
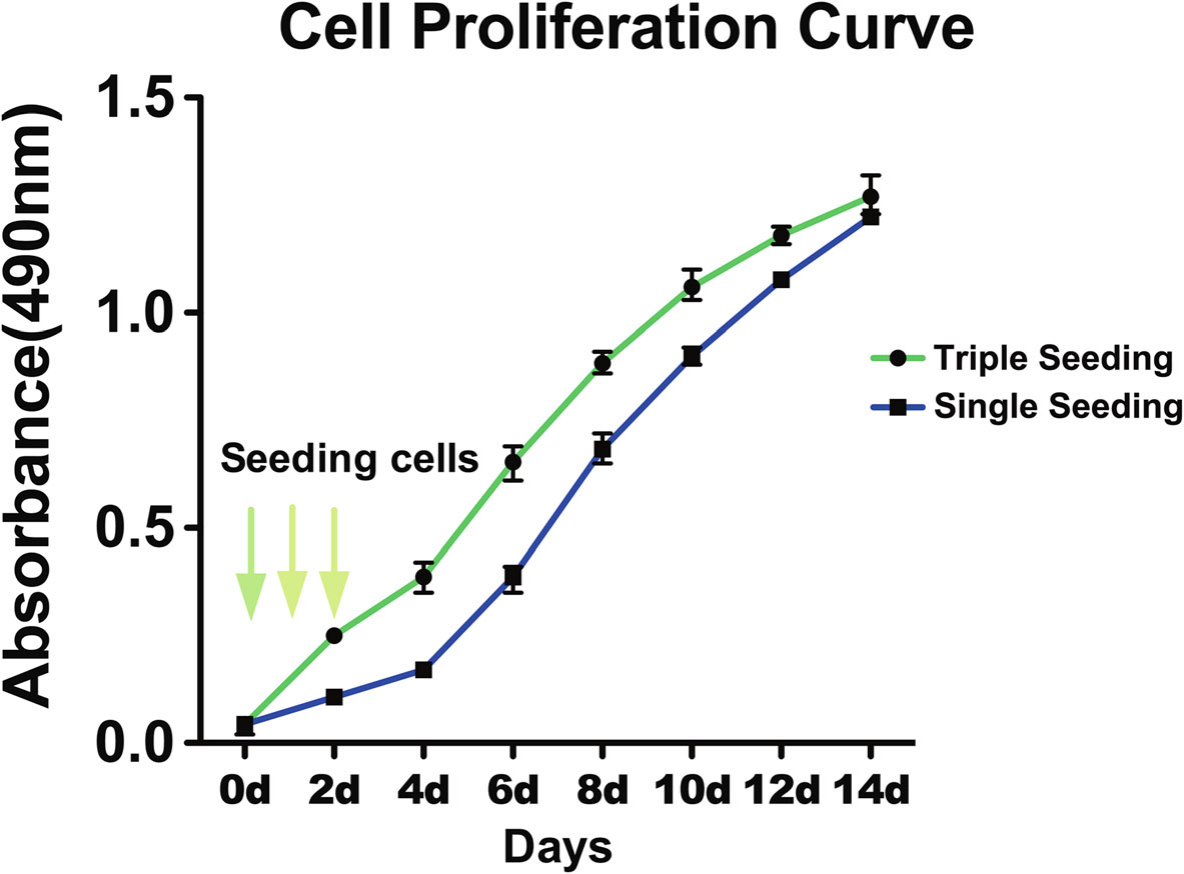
Cell proliferation curves of urothelially induced USC at different seeding cell concentrations. Cell growth pattern of single seeding was similar with that of triple seeding pattern. Dotted square showed at the 14th day the cell proliferation curve goes to almost same metric. Both groups were urine-derived stem cells cultured in urothelial-conditioned medium under dynamic culture environment. Notes: Single seeding = seeding cells only once, Triple seeding = seeding cells each at first 3 days

Triple-seeded USC presented significantly more cells on days 2, 4, 6, and 8 compared to single-seeded USC, but not on days 10, 12, and 14. Numbers of single-seeded USC grew up and caught up the number of triple-seeded USC on the last three time points in Fig. 5. Based on permeability analysis, it appears that single-seeding can form the similar barrier function as triple-seeding during 2-week induction culture, indicating that it requires time to fully develop tight junctions regardless of the number of cells present. However, urothelium tended to detach from transwell after 2-week induction.

**Fig. 6:**
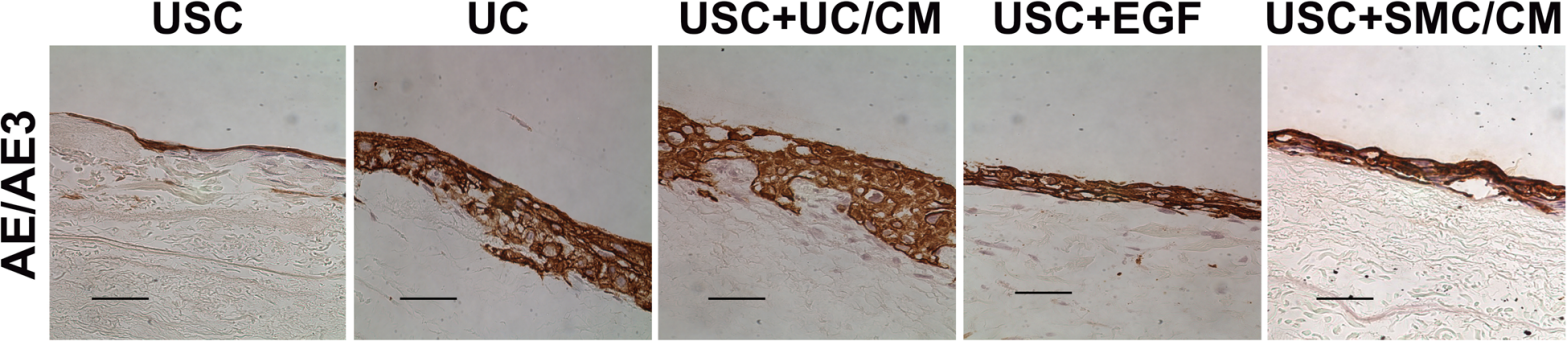
Effect of nature collagen matrix on multilayer formation of urothelium of UC-induced USC. Urothelially induced USC developed urothelium with five to seven cell layers stained positive for AE1/AE3 onto a SIS matrix under dynamic culture, which is similar to urothelial cells. In contrast, USC treated with EGF or SMC/CM grew thinner layer and USC alone formed a single layer. In addition, the induced USC penetrated into the porous SIS matrix to a great depth to form well adhesion structure of cell-matrix. Scale bar = 50 μm. Abbreviations: USC = urine-derived stem cells, UC = urothelial cells, SMC = smooth muscle cells, CM = conditioned medium, UC/CM = urothelium conditioned medium SMC/CM = smooth muscle cell-conditioned medium EGF = epidermal growth factor

In addition, triple-seeded USC formed a cell sheet on SIS that was five to six cells deep over 14 days under dynamic culture conditions (Fig. 6). No evidence of generation of internal necrotic regions was identified. This indicates that dynamic culture promotes USC proliferation and generates a healthy multilayer urothelium with a cellular microarchitecture. Immunochemical staining of USC-derived urothelium generated on decellularized SIS showed normal multilayered urothelial structures with well-organized luminal surface expression of AE1/AE3. These cells penetrated into the SIS with porous structure, providing good anchorage that limited the occurrence of detachment from the substrate (Fig. 6). Despite cells growing well on polyester membrane, the portions of the multilayer urothelium often separated from the rigid synthetic material were also washed away during immunocytochemical staining (not shown in figures). Remarkably, soft SIS matrix provides favorite subtract for multilayer urothelium formation in vitro. In order to obtain optimal histological slides with entire cell structures, the mechanical properties (such as hardness) of biomaterial should match to the soft cellular sheet for the histology processes to evaluate the microarchitecture of multiple layers of UC.

## Discussion

The overarching goal of this study is to develop an optimized method for increasing uroplakin expression and barrier function of human urothelium differentiated from USC by urothelial conditional media and dynamic culture system for potential use in urethra or bladder reconstruction. The underlying thought was that urothelium condition medium provides differentiation cues that guide USC to differentiate into urothelial cells in a more efficient manner than EGF alone. In addition, dynamic culture generated mechanical signals that promoted the formation of tight junctions. The present study demonstrated that the combination of urothelial conditional media and dynamic culture system provides preferred induction method for multiyear urothelium formation from stem cells in the urine.

Terminally differentiated urothelial cells are the most commonly used cells for the generation of multilayer urothelium intended for drug testing and use in urological tissue repair. However, patient-derived urothelial cells often cannot be obtained due to urethral stricture or bladder infection diseases or trauma. In addition, urothelial cells in certain patients may be affected by conditions such as bladder stones or other foreign bodies, presenting challenges in isolation and expansion of a sufficient cell population. Urothelial differentiation of USC offers a virtually limitless source of cells for model fabrication or tissue engineering. Several different types of stem cells, including MSC [10, 11], ESC [12], and iPSC [13], have been studied for urothelial cell differentiation. iPSC or ESC can give rise to functional urothelial cells, but the differentiation process is long and expensive. In addition, the differentiated cells carry the risk that rare, undifferentiated cells may retain the potential for teratoma formation. Although many reports describe urothelial cell differentiation from adult stem cells, most of these cells only display urothelial cell marker expression and do not measure barrier functionality. MSC give rise to very few urothelial cells with barrier function and the ability to form a multilayer structure. These cells have more limited differentiation potential than ESC, and typically only give rise to cell types within the same germ layer lineage. This produces enormous challenges when attempting to differentiate mesodermal MSC into endodermal urothelial cells.

USC are native to the urinary tract and can survive contact with urine, in the same manner as normal urothelial cells. USC are neither urothelial cells nor MSC, but they offer advantages over other multipotent cell types [24]. USC possess robust proliferation and multipotent differentiation potential [14, 19, 21]. Our previous studies have demonstrated that USC can be efficiently differentiated into urothelial cells in vitro and in vivo. Furthermore, USC isolation is trivial via a non-invasive method, and the cells expand extensively, in vitro [14, 20, 21]. Age and gender do not appear to impact the ability to harvest USC from urine.

Although urothelium possess multiple functions including sensory mechanisms (i.e., thermal, mechanical, and chemical sensors) and release chemical mediators, it serves mainly as a passive barrier to ions and solutes in urine. As a key protective attribute, urothelium barrier function is maintained by three structures: (1) uroplakin proteins in the apical cell membrane, (2) tight junctions localized between the superficial umbrella cells, (3) urothelial glycosaminoglycan (GAG) and proteoglycans, covering the umbrella cells [26]. Thus, uroplakin proteins, tight junction proteins, and histological or ultra-structure of cell/cell attachments are often assessed for evaluation of urothelial barrier function. Thus, urothelial barrier function is crucial in studies intended to identify treatments for a host of urinary tract diseases such as infection, interstitial cystitis, radiation cystitis, calculus formation, and cancer. Therefore, barrier function and histology are critical elements in the characterization of bioengineered urothelium. To date, most studies have shown that urothelial cells derived from adult stem cells displayed a certain amount of urothelial markers, but generally lack appropriate barrier function and histological structure [10, 11]. In this study, we demonstrate that urothelial cells differentiated from USC not only express urothelial specific markers, but also form tight junctions, arrange into an appropriate cellular architecture and maintain robust barrier function. Glycosaminoglycan layer is an important defense mechanism for the transitional epithelium mucosal surface of urinary tract. It allows adaptation to the constant exposure to urine and controls the permeability of urinary substances to the transitional cell. Urine appears necessary for induced stem cell to form elaboration of a GAG layer on the surface of multilayer urothelium [27]. As human urine was not used to induce GAG layer formation in this study, it is worthwhile to determine the GAG layer formation when differentiated USC are exposed to urine in a future study.

It is challenging to induce stem cell differentiation into functional cells because the molecular and mechanical factors that govern the differentiation program are not fully understood. Conditioned medium from cultured target cells is commonly used for induction of stem cells to differentiate renal tubule epithelial cells [27], germ cells [28], skeletal muscle cells, osteocytes [29], and chondrocytes [30]. Paracrine factors (i.e., growth factors, cytokines, tissue-specific genes, and microRNAs) that are released into the medium can regulate cell phenotype and are likely responsible for directing the differentiation of multipotent cells towards the intended cell type. Our previous studies have demonstrated that EGF alone may be used to promote differentiation of USC to cells that express urothelial cell markers [14, 19, 25]. The current study suggests that urothelial cell-conditioned medium provides a favorable source of inductive signals that initiate differentiation of USC towards the urothelial cell lineage. These urothelial-inductive factors remain incompletely characterized, although EGF is likely one of the major mediators of urothelial differentiation [14, 19, 25]. To understand the mechanism driving urothelial differentiation of USC, factors secreted by cultured urothelial cells would need to be separated by chromatography and characterized. The elucidation of these factors will need to be accomplished in future studies. This would provide a better understanding of the mechanisms driving urothelial cell differentiation and allow for the formation of even more physiologically normal USC-derived urothelium that could be used for generating in vitro models and for urological regenerative medicine therapies.

The urothelium is constantly exposed to fluctuating osmotic conditions, hydrostatic pressure, and mechanical strain, due to the dynamic environment produced by cyclic retention and voiding of urine. Our previous studies demonstrated that dynamic culture significantly enhanced cell growth and myogenic differentiation of bone marrow stromal cells (BMSC) by SMC-conditioned medium, as compare to static culture. By testing a series of dynamic conditions, it was determined that the optimal rotational rate for producing was 40 rpm [31]. However, dynamic culture in the absence of molecular differentiation cues promoted only cell proliferation but did not induce SMC differentiation from BMSC [22]. In this study, dynamic culture conditions significantly promoted barrier function in USC-derived urothelium, regardless of the differentiation protocol employed. The dynamic culture conditions most likely promoted cell/cell communications and promoted tight junction formation through increase mechanical stress. It appears that induced USC formed multipliers of urothelium so that number of cells did not continue increasing at the end of 14 days’ culture. These data suggest that dynamic culture conditions, mimicking the flow of urine, prompted the USC-derived UC to adopt a more normal phenotype and arrange into a normal urothelial cellular architecture.

The induction time frame is important for urothelial differentiation of stem cells. The state-of-the-art method for urothelial cell differentiation from USC uses EGF in static culture for 1 or 2 weeks. Our previous studies have shown that conditioned medium enhance urothelial cell differentiation and proliferation of rat urothelial progenitors [8], and dynamic culture promotes bladder SMC differentiation from USC [22] at different time periods. It is apparent that different time frames are required to induce stem cells to give rise to different cell types. Based on our previous studies, it takes approximately 4 weeks to induce human USC into skeletal muscle cells [14, 17, 20], 2 weeks for differentiation of SMC with contractile function [14, 20], and 1 to 2 weeks for endodermal differentiation with barrier function [14, 20]. However, many groups induce USC differentiation to UC that express urothelial markers over a single week. Our study indicates that the standard 1-week induction is not sufficient to give rise to a urothelial cell population with normal tight junctions (Fig. 2c). Of note, urothelium has tendency to detach from the substrate after 3 weeks of induction and tight junction is not fully developed at 1-week induction, indicating that 2 weeks is the optimal time frame for urothelial differentiation from USC.

One highly important characteristic of urothelium is its multilayer structure. Multilayer urothelium provides the bladder with the physical attributes required for urine storage (stretching) and voiding (contracting). Urothelial cells usually require a serum-free and calcium/magnesium-free culture condition [31]. Our previous studies and other laboratories have demonstrated that after urothelial cells reach confluence in serum-free keratinocyte medium, increasing extracellular calcium from 0.09 to 0.9 mM [32], or the addition of serum, promoted urothelial cell stratification and formation of tight junctions [14, 20, 21, 33]. Though not evaluated in the current study, calcium and serum might provide additional differentiation cues that could further enhance the functionality of the induced USC. In addition, natural collagen-based biomaterials possess beneficial properties, which promote urothelial cell growth, formation of a multilayer structure, and expression of urothelial cell functional markers. This multilayer urothelium derived from USC may provide an excellent model for research on human urothelial tissue and serve as a potential material for urological regenerative medicine therapies. It would be interesting to examine whether normal urothelium structures including three layers: basal, intermediate, and apical, form in the tissue engineered multilayer urothelium in a future study. Additionally, characterization of organelle structure, urothelial mechanical properties, and additional functional biomarkers needs to be conducted to confirm the functional potential of USC derived urothelium.

In addition, it is critical to consider interactions of the drug with both normal and diseased urothelium to improve the safety and efficiency of drugs delivered locally to the bladder. Thus, tissue-engineered urothelium also provides a convenient tool for studying these interactions, beside urological tissue repair. The development of disease-specific urothelium models based on the technologies described herein would increase our understanding of locally delivered bladder drug kinetics in both normal and diseased tissue.

## Conclusions

Tissue engineered urethra or bladder and in vitro bladder mucosa models for anti-cancer or interstitial cystitis drug development require urothelial cells for production. However, urothelial cells are often obtained by invasive bladder tissue biopsy. Although adult stem cells maintain urothelial differentiation potential, affecting this differentiation remains challenging. In this study, we have demonstrated that stem cells present in urine can efficiently differentiate into urothelial cells with robust barrier function that form multilayered structures similar to normal urothelium. USC-derived urothelium induced by urothelial cell-conditioned medium showed multiple functional improvements over previous differentiation methods. Urothelium generated from patient-derived USC would provide an excellent platform for the study of mechanisms underlying urological diseases including interstitial cystitis, overactive, neurogenic, or obstructive bladder and provide a testbed for the development of therapies to treat these diseases. In addition, induced urothelium can be used in evaluating the impact of pharmacological treatment on urothelial barrier function in both normal bladder and in bladder that has been compromised by diseases. Furthermore, multiple layer urothelium formed on a natural collagen-based matrix may be used for the reconstruction of urological tissues that have been damaged by trauma or disease.

## Additional files

Additional file 1: **Table S1.** Primers for real-time PCR used in this study. (DOCX 13 kb)

Additional file 2: **Table S2.** Antibodies used in this study. (DOCX 14 kb)

Additional file 3: **Table S3.** Percentage of USC expressing urothelial cell markers 2 weeks after urothelial induction assessed by immunofluorescence. (DOCX 14 kb)

Additional file 4: **Table S4.** Percentage of USC expressing tight junction markers 2 weeks after urothelial induction assessed by immunofluorescence. (DOCX 16 kb)

## Abbreviations

BMSC: Bone marrow stromal cells
BSA: Bovine serum albumin
CM: Conditioned medium
DAPI: Diamidino-2-phenylindole
DMEM: Dulbecco’s modified Eagle’s medium
EFM: Embryo fibroblast medium
EGF: Epidermal growth factor
ESC: Embryonic stem cells
FBS: Fetal bovine serum
GAG: Glycosaminoglycan
iPSC: Induced pluripotent stem cells
KSFM: Keratinocyte serum-free medium
MSC: Mesenchymal stem cells
PAA: Peracetic acid
PBS: Phosphate buffered saline
SIS: Small intestinal submucosa
SMC: Smooth muscle cells
TEM: Transmission electron microscopy
UC: Urothelial cells
USC: Urine-derived stem cells
