# Supplementary material for: Urothelium with barrier function differentiated from human urine-derived stem cells for potential use in urinary tract reconstruction
Source: Stem Cell Res Ther. 2018 Nov 8;9:304. doi: 10.1186/s13287-018-1035-6 (PMC6225683; doi:10.1186/s13287-018-1035-6)
Supplement: Supplementary file 1 — Table S1. Primers for real-time PCR used in this study. (DOCX 13 kb) [file 13287_2018_1035_MOESM1_ESM.docx]

| **Table S1.** Primers for real-time PCR used in this study | |
| --- | --- |
| Primary Antibody | Catalog# |
| CK-20 | Hs00300643_m1 |
| AE1(SLC4A1) | Hs00978607_g1 |
| AE3(SLC4A3) | Hs00192595_m1 |
| UPIa | Hs00199638_m1 |
| UPIII | Hs00199590_m1 |
| E-cadherin | Hs01023894_m1 |
| Cingulin | Hs00430426_m1 |
| ZO1 | Hs01551861_m1 |
| ZO2 | Hs00910543_m1 |
| GAPDH | NM_002046.3 |
| *All primers obtained from Applied Biosystems, Foster City, CA* | |
