# Supplementary material for: Urothelium with barrier function differentiated from human urine-derived stem cells for potential use in urinary tract reconstruction
Source: Stem Cell Res Ther. 2018 Nov 8;9:304. doi: 10.1186/s13287-018-1035-6 (PMC6225683; doi:10.1186/s13287-018-1035-6)
Supplement: Supplementary file 2 — Table S2. Antibodies used in this study. (DOCX 14 kb) [file 13287_2018_1035_MOESM2_ESM.docx]

| **Table S2.** Antibodies used in this study | | | | | | |
| --- | --- | --- | --- | --- | --- | --- |
|  | Western Blotting | | | Immunofluorescence | | |
|  | Host | Dilution | Company | Host | Dilution | Company |
| CK-20 | Goat | 1:200 | Santa Cruz  Sc-17112 | Goat | 1:50 | Santa Cruz  Sc-17112 |
| AE1/AE3 | Mouse | 1:400 | Santa Cruz  Sc-81714 | Mouse | 1:400 | Dako  GA053 |
| UPIa | Goat | 1:100 | Santa Cruz  Sc-15173 | Goat | 1:50 | Santa Cruz  Sc-15173 |
| UPIII | Mouse | 1:1000 | Abcam  Ab-78196 | Mouse | 1:100 | Abcam  Ab-78196 |
| E-cadherin | Mouse | 1:1000 | Abcam  Ab-1416 | Mouse | 1:200 | Abcam  Ab-1416 |
| Cingulin | Rabbit | 1:200 | Santa Cruz  Sc-66831 | Rabbit | 1:50 | Santa Cruz  Sc-66831 |
| ZO1 | Rabbit | 1:500 | Abcam  Ab-59720 | Mouse | 1:400 | Life Technology  33-9100 |
| ZO2 | Rabbit | 1:500 | Life  Technology  38-9100 | Rabbit | 1:400 | Life Technology  38-9100 |
| β-actin | Mouse | 1:10000 | Sigma-Aldrich  A2228 |  |  |  |
