# Supplementary material for: Urothelium with barrier function differentiated from human urine-derived stem cells for potential use in urinary tract reconstruction
Source: Stem Cell Res Ther. 2018 Nov 8;9:304. doi: 10.1186/s13287-018-1035-6 (PMC6225683; doi:10.1186/s13287-018-1035-6)
Supplement: Supplementary file 3 — Table S3. Percentage of USC expressing urothelial cell markers 2 weeks after urothelial induction assessed by immunofluorescence. (DOCX 14 kb) [file 13287_2018_1035_MOESM3_ESM.docx]

|  | **UPIa** | **UPIII** | **AE1/AE3** | **CK20** |
| --- | --- | --- | --- | --- |
| G1 USC | 3.4±2.2 | 2.1±1.6 | 18.2±2.9 | 2.7±1.8 |
| G2 UC | 32.6±3.6 | 39.3±5.0 | 94.5±3.7 | 71.2±14.3 |
| G3 USC+UC/CM | 31.9±4.6 | 34.5±6.1 | 72.6±8.5 | 42.2±8.4 |
| G4 USC+EGF | 16.3±3.2 | 18.2±3.4 | 37.1±5.4 | 8.6±5.8 |
| G5 USC+SMC/CM | 5.9±4.8 | 7.7±3.8 | 21.4±4.2 | 7.4±2.1 |

**Table S3.** Percentage of USC expressing urothelial cell markers 2 weeks after urothelial induction assessed by immunofluorescence

**Notes:** *The data are represented as Mean ± SEM.*

**Abbreviations**: *UPIa=Uroplakin Ia, UPIII= Uroplakin III, USC=urine derived stem cells, UC= urothelial cells, SMC=smooth muscle cells, CM=conditioned medium, UC/CM= Urothelium conditioned medium SMC/CM=Smooth muscle cells conditioned medium EGF=epidermal growth factor.*
