# Supplementary material for: Urothelium with barrier function differentiated from human urine-derived stem cells for potential use in urinary tract reconstruction
Source: Stem Cell Res Ther. 2018 Nov 8;9:304. doi: 10.1186/s13287-018-1035-6 (PMC6225683; doi:10.1186/s13287-018-1035-6)
Supplement: Supplementary file 4 — Table S4. Percentage of USC expressing tight junction markers 2 weeks after urothelial induction assessed by immunofluorescence. (DOCX 16 kb) [file 13287_2018_1035_MOESM4_ESM.docx]

| **Table S4.** Percentage of USC expressing tight junction markers 2 weeks after urothelial induction assessed by immunofluorescence | | | | |
| --- | --- | --- | --- | --- |
|  | **ZO1** | **ZO2** | **E-cadherin** | **Cingulin** |
| G1 USC | 4.5±2.4 | 11.3±3.6 | 10.4±5.3 | 6.6±4.6 |
| G2 UC | 58.4±7.8 | 86.2±9.6 | 74.2±10.8 | 79.3±6.8 |
| G3 USC+UC/CM | 55.7±5.3 | 87.1±12.0 | 71.9±4.2 | 64.5±7.5 |
| G4 USC+ EGF | 23.2±3.1 | 24.6±5.5 | 32.4±2.8 | 35.3±9.7 |
| G5 USC+SMC/CM | 18.8±6.0 | 17.4±3.3 | 23.2±5.0 | 20.3±3.7 |
| **Notes:** *The data are represented as Mean ± SEM.*  **Abbreviations**: *USC=urine derived stem cells, UC= urothelial cells, SMC=smooth muscle cells, CM=conditioned medium, UC/CM= Urothelium conditioned medium SMC/CM=Smooth muscle cells conditioned medium EGF=epidermal growth factor.* | | | | |
